# Scale-up of the Diactive-1 mHealth program integrating tailored resistance training as an educational strategy for self-management in children and adolescents with type 1 diabetes: protocol for an 11-hospital multicenter randomized controlled trial

**DOI:** 10.3389/fendo.2026.1913568

**Published:** 2026-08-14

**Authors:** Amaia Sánchez-Arlegui, Jacinto Muñoz-Pardeza, Ignacio Hormazábal-Aguayo, Sonia Abio-Abero, María Laura Bertholt-Zuber, Pablo Alonso-Rubio, Elisabet Burillo-Sánchez, María J. Chueca-Guindulain, Claudia Cifuentes-Zamalloa, Sofía Congost-Marín, Ignacio Díez-López, Marta Ferrer-Lozano, Patricia García-Navas, Lara Gloria González-García, Belén Huidobro-Fernández, Paula Lalaguna-Mallada, María Teresa Llorente-Cereza, Sandra Maeso-Mendez, Isolina Riaño-Galán, María Ruiz-del Campo, Oihane Salcedo Fresneda, Marta Vara-Callau, Amaia Vela-Desojo, Yasmin Ezzatvar, Antonio García-Hermoso

**Affiliations:** 1 Public Healthcare Service of Navarra-Osasunbidea (SNS-O), Universidad Pública de Navarra (UPNA), Pamplona, Spain; 2 Navarrabiomed, Hospital Universitario de Navarra, Universidad Pública de Navarra (UPNA), Instituto de Investigación Sanitaria de Navarra (IdiSNA), Pamplona, Spain; 3 Office of the Vice-Rector for Research and Graduate Studies, Universidad de La Serena, La Serena, Chile; 4 Pediatrics Service, Hospital San Jorge, Huesca, Spain; 5 Pediatric Endocrinology Unit, Hospital Universitario Marqués de Valdecilla, Santander, Spain; 6 Pediatric Endocrinology Unit, Hospital Universitario de Navarra, Instituto de Investigación Sanitaria de Navarra (IdiSNA), Pamplona, Spain; 7 Pediatric Endocrinology Unit, Hospital Universitario Cruces, Barakaldo, Spain; 8 Pediatric Endocrinology Unit, Hospital Clínico Universitario Lozano Blesa, Zaragoza, Spain; 9 Pediatrics Service, Hospital de Araba, Vitoria, Spain; 10 Pediatric Endocrinology Unit, Hospital Infantil Miguel Servet, Zaragoza, Spain; 11 Pediatrics Service, Hospital San Pedro, Logroño, Spain; 12 Pediatrics Service, Hospital de Cabueñes, Gijón, Spain; 13 Pediatrics Service, Hospital de Barbastro, Huesca, Spain; 14 Pediatric Endocrinology Unit, Hospital Universitario Central de Asturias, Oviedo, Spain; 15 Lifestyle factors with impact on Ageing and overall Health (LAH) Research Group, Departamento de Enfermería, Universidad de València, Valencia, Spain; 16 Vicerrectoría de Investigación y Postgrado, Universidad de Los Lagos, Osorno, Chile

**Keywords:** digital health, education, mHealth, strength training, type 1 diabetes

## Abstract

**Introduction:**

Physical activity is strongly recommended for youth with type 1 diabetes, yet many children and adolescents do not meet current exercise guidelines, partly because of fear of hypoglycemia. Resistance training may offer metabolic and musculoskeletal benefits, but its implementation in routine pediatric diabetes care remains limited. Diactive-1 is an mHealth program designed to deliver tailored resistance-training sessions combined with glucose-management educational support. This multicenter trial aims to evaluate the effectiveness of Diactive-1 (v2.0) in improving hangrip strength in children and adolescents with type 1 diabetes.

**Methods:**

This study is a 12-week, parallel-group, multicenter randomized controlled trial conducted across 11 Spanish pediatric endocrinology units. The target sample size is 158 children and adolescents aged 10–18 years with type 1 diabetes, with 79 participants allocated to each group. Participants will be randomized to either the Diactive-1 intervention or usual clinical care. The intervention group will use the Diactive-1 (v2.0) app, which provides individualized resistance-training programs, glucose-related educational prompts, safety thresholds for exercise, and engagement strategies such as gamification. The primary outcome will be the change in age- and sex-standardized handgrip strength z-score from baseline to 12 weeks. Secondary outcomes will include glycemic stability, insulin requirements, cardiometabolic markers, physical literacy, quality of life, psychological well-being, app usability, and intervention engagement. Analyses will follow the intention-to-treat principle using mixed-effects models.

**Expected outcomes and discussion:**

This trial will assess whether a scalable mHealth-supported resistance-training program can improve muscular strength and support diabetes self-management in routine pediatric care. By testing Diactive-1 across multiple hospitals and real-world settings, the study will provide evidence on the external validity, feasibility, and potential clinical integration of digitally supported exercise education for youth with type 1 diabetes.

**Ethics and dissemination:**

Ethical approval was granted by the Research Ethics Committee for Medicinal Products of the Government of Navarra. Written informed consent will be obtained from parents or legal guardians, with age-appropriate assent from minors. Findings will be disseminated through scientific conferences, peer-reviewed publications, and summaries shared with participating families.

**Clinical Trial Registration:**

https://clinicaltrials.gov/study/NCT07290868, identifier NCT07290868.

## 1 Introduction

In 2024, the incidence of type 1 diabetes mellitus, a disease caused by immune-mediated destruction of pancreatic β-cells leading to a lack of endogenous insulin production (1), reached 219,000 new cases among children and adolescents worldwide (2), with a rate of 14.07 per 100,000 (3). This condition results in elevated blood glucose levels, known as hyperglycemia, which over time increases the risk of both macrovascular and microvascular complications (4). Indeed, it is estimated that 1 in 10 youth already exhibit microvascular impairments (5), although other complications, such as loss of muscle mass and function, should also be considered (6). Beyond the physical domain, young individuals face daily self-management demands related to type 1 diabetes management that may compromise emotional health (7). Therefore, daily diabetes care warrants close attention, as glycemic stability may have implications even for quality of life (8).

The primary therapy for type 1 diabetes is exogenous insulin administration; however, this approach is imperfect, as it contributes to the risk of hypoglycemia, defined as low blood glucose levels (9). Therefore, non-pharmacological strategies that support safe self-management in youth with type 1 diabetes should also be considered (10). Among these, physical exercise is recognized as a safe and effective adjunctive strategy for optimizing type 1 diabetes management (11). In particular, resistance training is clinically relevant because muscle-strengthening activities are recommended for children and adolescents with type 1 diabetes, yet this modality remains less studied than aerobic exercise (12). Despite the benefits of exercise, youth with type 1 diabetes tend to be more sedentary than their apparently healthy peers (13), with one of the primary barriers being fear of hypoglycemia (14). However, the literature suggests that this concern may partly reflect inadequate insulin dose adjustments around exercise rather than exercise intensity itself (15). Recent technological advances, including continuous glucose monitoring (CGM) and increasingly flexible insulin delivery systems, may help improve glycemic stability while reducing the burden of self-management (16, 17). Nevertheless, the interaction between these technologies and exercise requires careful consideration; accordingly, recent guidelines address glycemic management using CGM and automated insulin delivery (AID) systems in the context of exercise (18, 19).

In the 2026 Standards of Care in Diabetes, the American Diabetes Association (ADA) continues to recommend ≥60 minutes per day of moderate-to-vigorous physical activity and ≥3 days per week of muscle-strengthening activities for children and adolescents, recognizing exercise for the first time as an effective therapeutic strategy (20). However, a key challenge lies in enabling youth with type 1 diabetes to translate guideline-based recommendations into daily practice while understanding their individual glycemic responses to exercise. To address this gap, Diactive-1 was developed as an mHealth tool that leverages the widespread use of smartphones worldwide (21) to deliver tailored resistance training programs, responding to the limited evidence on the isolated effects of this modality (22), while incorporating glycemic monitoring around exercise to provide evidence-based recommendations (18). Our previous randomized clinical trial (RCT) conducted in Navarre, Spain, involving 62 youth with type 1 diabetes showed high usability of Diactive-1, with a score of 4.33 out of 5.00 (23), and demonstrated that participants allocated to the intervention group maintained glycemic stability while achieving significant reductions in daily insulin dose at 12 and 24 weeks compared with usual clinical care, with no reported hypoglycemic events (10). These benefits were accompanied by improvements in muscular strength, lean mass, and bone mineral content (24). Thus, Diactive-1 may have potential as an adjunctive tool to standard treatment by empowering youth to optimize daily type 1 diabetes management. However, the initial RCT was conducted in a single clinical context with a limited sample size, and the effectiveness of Diactive-1 across other hospital settings and clinical workflows remains uncertain. This could be addressed by scaling up Diactive-1 across multiple hospitals, incorporating an updated algorithm, refining the training program, and enhancing usability to promote greater engagement. Therefore, the primary aim of this multicenter randomized controlled trial is to evaluate the effectiveness of Diactive-1 (v2.0) in improving handgrip strength in children and adolescents with type 1 diabetes. Secondary aims are to examine its effects on glycemic stability, insulin requirements, cardiometabolic markers, physical literacy, quality of life, psychological well-being, and app-related engagement outcomes.

## 2 Materials and methods

### 2.1 Trial design

This study is designed as a 12-week, parallel-group (i.e., intervention group and usual clinical care group), multicenter RCT conducted across eleven pediatric endocrinology units in Spanish hospitals: *Universitario de Navarra* (Pamplona, Navarra), *Universitario Marqués de Valdecilla* (Santander, Cantabria), *Cabueñes* (Gijón, Asturias), *Universitario Central de Asturias* (Oviedo, Asturias), *Universitario de Cruces* (Barakaldo, País Vasco), *Universitario Araba* (Vitoria-Gasteiz, País Vasco), *San Millán-San Pedro* (Logroño, La Rioja), *General San Jorge* (Huesca, Aragón), *de Barbastro* (Barbastro, Aragón), *Clínico Universitario Lozano de Blesa* (Zaragoza, Aragón), and *Universitario Miguel Servet* (Zaragoza, Aragón). The central coordinating site for the project will be *Navarrabiomed* Biomedical Research Center (Pamplona, Navarra) from which the current RCT has been registered on ClinicalTrials.gov (Reg. ID: NCT07290868). This protocol has been developed in accordance with the Standard Protocol Items: Recommendations for Interventional Trials (SPIRIT) statement for RCT protocols (25).

### 2.2 Study setting

The RCT will be conducted in a hybrid setting combining routine clinical care in hospital-based pediatric endocrinology outpatient clinics with real-world daily environments. Baseline and post-intervention assessments will be performed during routine outpatient visits by healthcare professionals trained in standardized assessment procedures, and data will be recorded through the Diactive-1 clinical web-based platform. The 12-week duration is supported by preliminary Diactive-1 findings showing relevant effects within this timeframe (10, 24). The intervention will be delivered through the Diactive-1 mHealth app in participants’ daily environments, distinguishing it from usual clinical care.

The project was initially approved by the Medical Research Ethics Committee of Navarra (PS_2025/5), approved on March 25, 2025, and has undergone additional review by the ethics committees of each hospital. The study will adhere to the ethical guidelines of the Declaration of Helsinki, the Singapore Statement, and the Oviedo Convention on Human Rights and Biomedicine.

### 2.3 Eligibility criteria

This study will include children and adolescents aged 10 to 18 years with a diagnosis of type 1 diabetes mellitus of at least 6 months’ duration, with insulin requirements >0.5 U/kg/day and HbA1c >6% (26). Additional inclusion criteria are: (i) current treatment with multiple daily injections (MDI) or insulin pump therapy; (ii) use of CGM; (iii) availability to participate in the study using the Diactive-1 mHealth application; (iv) provision of written informed consent authorizing participation, signed by both the participants and their legal guardians; and (v) ability to understand Spanish or English, the languages supported by the application. Exclusion criteria are: (i) presence of comorbidities that limit the ability to perform physical activity during the study period; (ii) lack of access to a compatible device, either personal or loaned, with Android or iOS operating systems on which the Diactive-1 application can be installed; and (iii) lack of internet connectivity at the time of application use, including Wi-Fi, mobile data, or international roaming.

### 2.4 Sample size and recruitment

Based on preliminary 12-week intervention data from the previous Diactive-1 trial, the between-group difference in handgrip strength z-score was 0.24 units, corresponding to an approximate standardized effect size of Cohen’s d = 0.56 (10). Using G*Power for an independent-samples comparison with a two-sided α = 0.05, 134 evaluable participants (67 per group) would provide 89.6% power, rounded to approximately 90% (27). After allowing for an anticipated attrition rate of 15%, the recruitment target was set at 158 children and adolescents in total, with 79 participants allocated to the intervention group and 79 to the usual-care group (28). Sampling will be extended until the end of the recruitment period, incorporating as many participants as possible.

Because participants will be individually randomized within hospitals, this is not a cluster-randomized trial. Nevertheless, hospital-level effects will be accounted for in the planned mixed-effects analyses by including hospital as a random effect. As the actual hospital-level ICC is not yet known, a conservative *a priori* sensitivity analysis was conducted using the design effect DE = 1+(m−1)×ICC. Assuming a mean hospital size of approximately 14.5 participants and 15% attrition, ICC values of 0.01, 0.02, and 0.05 corresponded to effective sample sizes of approximately 120, 107, and 81 participants, with estimated powers of approximately 86%, 82%, and 70%, respectively. Thus, the planned recruitment is expected to retain more than 80% power under hospital-level ICC values of up to 0.02, whereas greater clustering would result in reduced power. Recruitment may continue beyond the target sample until the end of the prespecified recruitment period.

Recruitment will take place during outpatient visits in the pediatric endocrinology units of the participating hospitals, where healthcare professionals will inform eligible participants about the study protocol, assessments, the importance of randomization, and the potential risks and benefits, in accordance with the participant information sheet. Written informed consent will subsequently be obtained, with one copy retained by the healthcare staff in a physical file and another provided to the participants or their legal guardians. All individuals meeting the inclusion criteria will be eligible to participate to minimize selection bias, with enrollment continuing until the target sample size is reached and potentially exceeded through September 30, 2026.

### 2.5 Randomization, allocation concealment, and blinding

The randomization sequence assigning participants to the study groups (i.e., exercise intervention group and usual care wait-list control group) will be generated by a computerized algorithm with no human access and implemented via the centralized Diactive-1 web-based platform. The algorithm will use a non-stratified randomization scheme with small, randomly varying even block sizes (i.e., 2, 4, 6, and 8 participants). Each block will contain an equal allocation ratio between the two groups (50%–50%), with the order of assignments within each block randomly permuted, thereby ensuring balance between groups throughout recruitment while minimizing predictability of the sequence. Because recruitment per hospital may vary, randomization will be centralized and non-stratified, with hospital-level clustering accounted for in the mixed-effects models. To ensure allocation concealment, randomization will be automatically performed after completion of the baseline clinical visit and entry of the corresponding clinical data, before the 7-day diabetes-related data-collection period.

Participants and healthcare professionals cannot be blinded due to the nature of the intervention. Outcome processing and statistical analyses will be performed using coded group allocation whenever feasible. The statistical analyst will remain blinded to group identity until the primary analyses have been completed. All hospitals and participants registered on the Diactive-1 web-based platform will be coded using alphanumeric identifiers.

### 2.6 The Diactive-1 web platform

The Diactive-1 web platform (version 1.0) is a secure software system developed to connect the participating pediatric endocrinology units with the project coordination team. The platform supports three user roles: healthcare professionals, participants, and the coordination team. Access will be role-based, password-protected, and restricted to the minimum information required for each user profile.

Upon initial access, healthcare professionals will be required to complete tutorial videos covering platform functionality, CGM and insulin pump data extraction, study protocol procedures, standardized handgrip dynamometry, and anthropometric assessment. Subsequent access will provide a hospital-restricted dashboard displaying participant-level data only for participants recruited at that hospital. Locally identifiable information required for clinical follow-up and participant management will be accessible only to authorized healthcare professionals at the recruiting site. Research data will be linked to a coded participant identifier.

A dedicated module will enable participant registration and data entry, including contact/access information, date of birth, biological sex, Tanner stage, anthropometry, disease-related clinical variables, insulin delivery modality, pump and CGM system information, and handgrip strength. Direct identifiers, such as name and email address, will be stored separately from coded research data and will not be routinely accessible to the coordination team unless strictly required for study management or participant safety.

Healthcare professionals will also be able to upload de-identified or pseudonymized PDF files related to diabetes management, including CGM and AID reports. Before upload, direct identifiers will be removed whenever possible. Once baseline data have been entered and verified, the platform will interface with the randomization module described in Section 2.5 and generate group-specific access instructions through the secure platform.

Upon first access, participants will be required to view tutorial videos relevant to their study procedures. Participants allocated to the intervention group will additionally receive instructions on using the Diactive-1 mHealth app, including breathing techniques during movement, movement patterns, core muscle activation, and correct exercise execution. Participants will only have access to their own study-related content.

The coordination team interface will display a centralized list of coded participants, including creation date, affiliated hospital, questionnaire completion status, remaining study days, and follow-up status. The coordination team will not have routine access to direct participant identifiers. Any data correction after verification will require an authorized, documented, and audit-trailed amendment rather than unrestricted editing.

### 2.7 Procedure for unblinding if needed

The coordination team may unblind the data when necessary to ensure participant safety, following a medical request or a formal request from the ethics committee. In such cases, the principal investigator will request unblinding through the independent data manager using the Diactive-1 system. Only strictly necessary information will be disclosed, and all cases will be thoroughly documented and reported to the relevant ethics committee.

### 2.8 The Diactive-1 mHealth intervention

**Figure 1:**
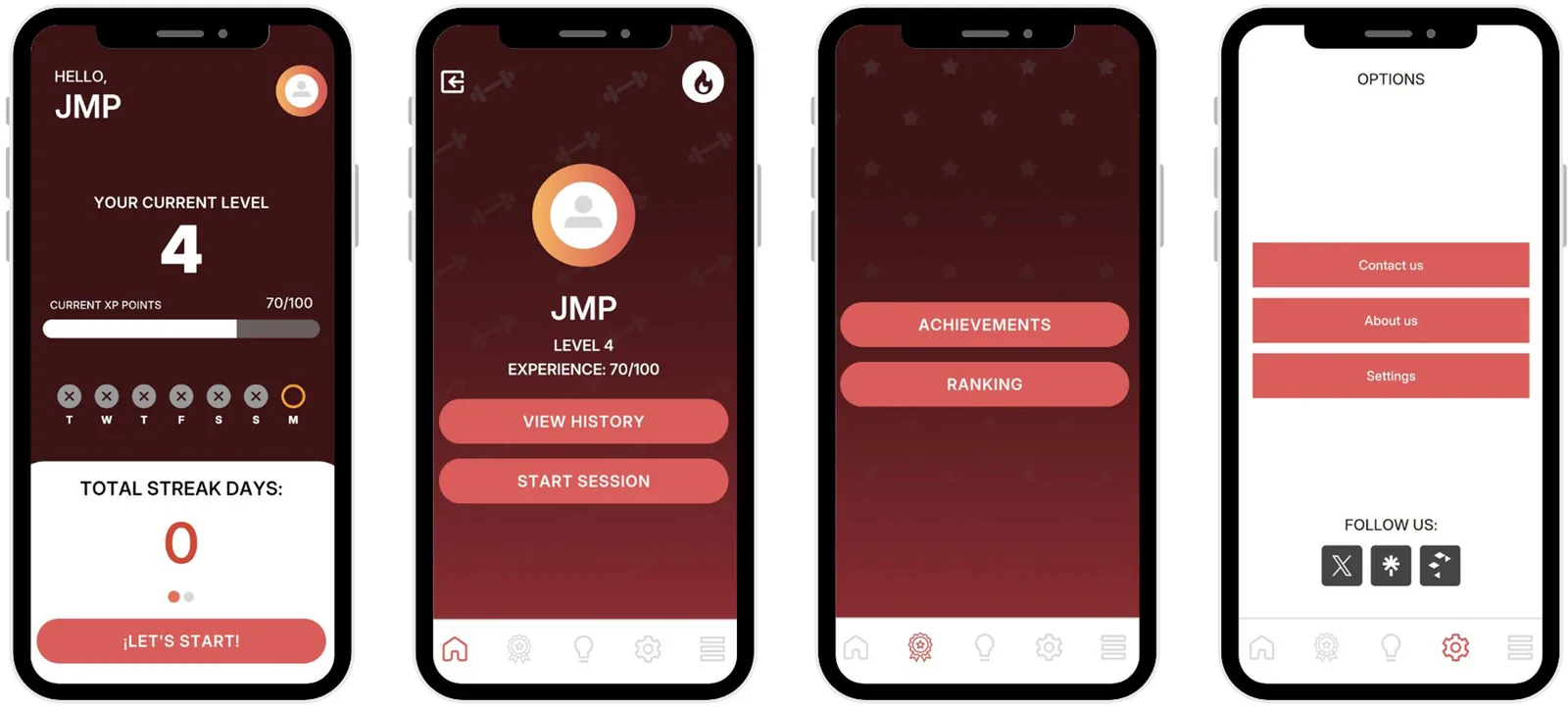
Diactive-1 app interface and main navigation screens.

The central component of this 12-week RCT is the mobile application used by the intervention group during the study period. Diactive-1 is an mHealth application that delivers tailored resistance-training programs comprising more than 350 exercises and is intended to support, but not replace, exercise professionals. **Figure 1** presents the main app interface and user navigation screens. This line of research focuses on implementing innovative physical exercise-based educational strategies for individuals with chronic conditions, aiming to enhance self-management capacity through empowerment-oriented interventions, in accordance with guidelines from the International Society for Pediatric and Adolescent Diabetes (ISPAD) (29). By reducing uncertainty and facilitating real-time decision-making, Diactive-1 aims to support diabetes self-management. Diactive-1 should therefore be considered a multicomponent self-management package, and the trial will estimate the combined effect of its tailored resistance-training prescription, glucose-management education, safety thresholds, tutorials, and gamification rather than the isolated effect of resistance training.

#### 2.8.1 Development of the study

The project was conceived by a multidisciplinary team comprising physicians, nutritionists, psychologists, physiotherapists, educators, exercise professionals, and innovation specialists. The Diactive-1 web platform and mobile application were developed by software engineers and graphic designers in collaboration with the multidisciplinary research team. Previous usability and preliminary effectiveness findings informed the refinement of Diactive-1 (v2.0), including updates to the training algorithm, educational content, and user-engagement features.

#### 2.8.2 Content and use of the usual clinical care group

Participants allocated to the usual care group will receive standard multidisciplinary diabetes management within the public healthcare system, including insulin therapy, glucose monitoring, structured diabetes education, and routine follow-up visits. Habitual physical activity, such as school-based physical education or pre-existing sports participation, will be permitted throughout the study. After completion of the 12-week post-intervention assessment and the subsequent 7-day post-visit diabetes-related data collection, participants in the usual care group will be offered access to the Diactive-1 mHealth application. Any clinically indicated modifications to diabetes treatment or technology will be made at the discretion of the treating clinician and documented accordingly.

#### 2.8.3 Content and use of the intervention group

Participants will use the Diactive-1 app (v.2.0), which delivers resistance-training programs that can be performed in any setting (e.g., home, park, or school). Programs are stratified into three levels (i.e., low [≤ 20^th^], medium [21^st^–79^th^], and high [≥80^th^]) according to baseline handgrip-strength percentiles derived from age- and sex-specific European FitBack^©^ reference values for children and adolescents (30). The training algorithm incorporates progressive overload within each level. Intensity increases across levels through greater technical complexity, progressing from less functional to more functional movement patterns, while volume increases through prespecified changes in the number of exercises per session, sets, repetitions, and recovery intervals.

**Figure 2:**
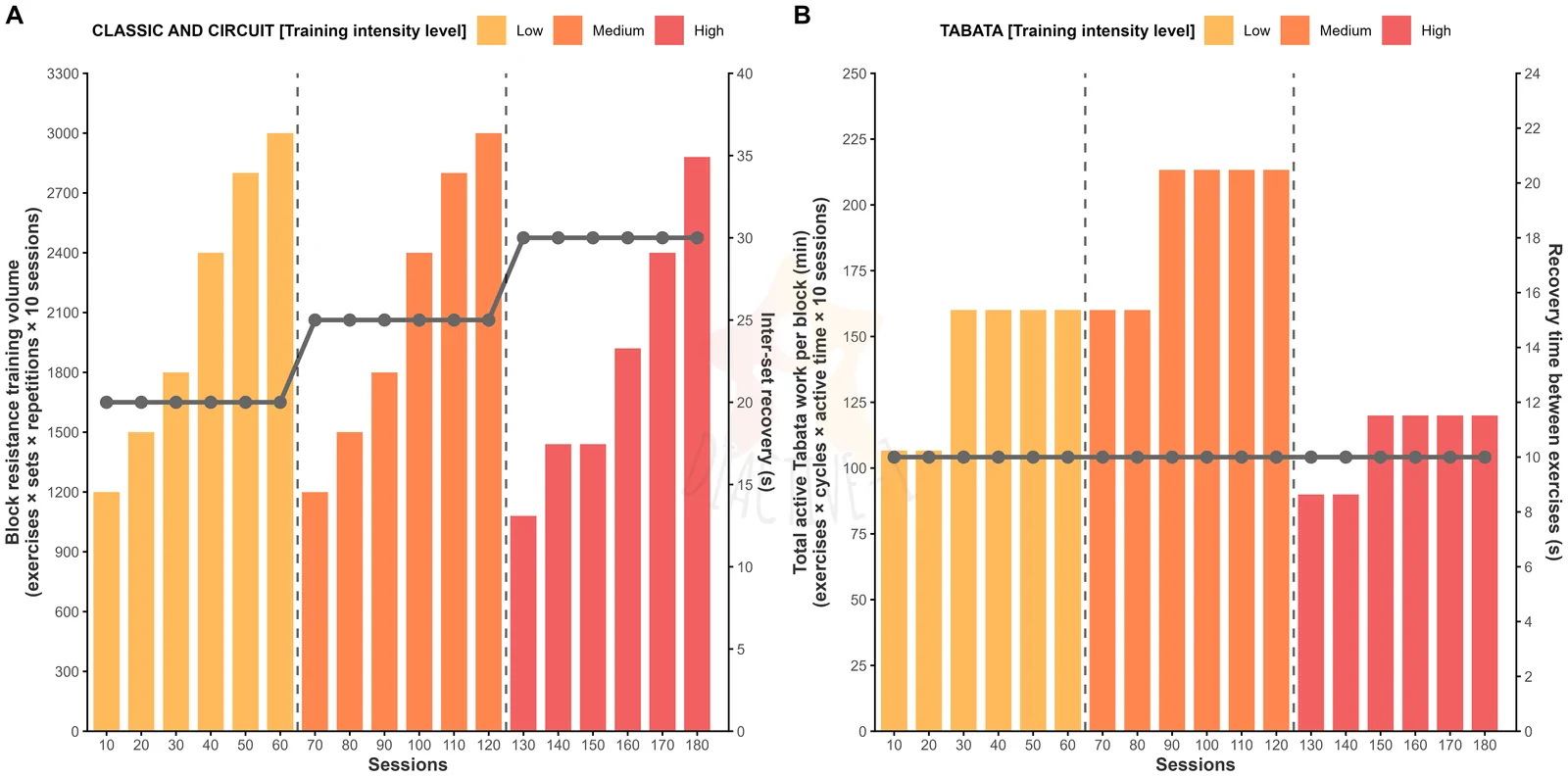
Programmed progression of training volume and recovery intervals for the classic, circuit, and Tabata resistance-training modalities in Diactive-1: **(A)** classic and circuit training; **(B)** Tabata training.

Low- and medium-level sessions include five exercises arranged in a pyramidal sequence alternating body segments to reduce localized fatigue and support adaptation during the first technical progression across levels (i.e., core, lower limb, upper limb, lower limb, core). High-level sessions include six exercises grouped by body segment to increase internal load (i.e., two core, two upper-limb, and two lower-limb exercises). Within each session, exercises with similar movement patterns will not be repeated. Inter-exercise recovery remains constant throughout the program (40 seconds), while inter-set recovery increases across training levels (20, 25, and 30 seconds) to support tolerance to increasing neuromuscular demands. Load progression is organized into sequential ten-session blocks, beginning with an increase in repetitions (from 6–8 to 12–15) per set. After participants reach the upper repetition range, the number of sets per exercise increases (from 3 to 4), with a temporary reduction in repetitions to preserve effort tolerance; repetitions are then progressively increased within the new set structure. This progression applies to both classic and circuit training modes. Participants may also select a short-duration Tabata mode, consisting of repeated sets alternating with 20 seconds of exercise, during which they perform as many repetitions as possible, with 10 seconds of recovery. Once all programmed exercises within a set block are completed, the block ends and the sequence restarts until the prescribed number of blocks is completed. **Figure 2** shows the programmed load progression for each training modality.

Diactive-1 (v.2.0) requires participants to enter glucose values before and after each training session. These values are used to provide educational messages based on CGM readings (18). In this updated version, additional messages are provided for participants using the Medtronic MiniMed 780G automated insulin delivery system (19), reflecting its frequent use in the participating Spanish clinical settings. These messages are intended to support type 1 diabetes self-management and the safe completion of the corresponding resistance-training session. In general, messages for hyperglycemia focus on insulin-dose adjustment, while messages for hypoglycemia focus on rapid-acting carbohydrate intake according to the participant’s body mass. Specifically, training is not permitted when glucose is <70 mg/dL (<3.9 mmol/L) or when glucose is >270 mg/dL (>15.0 mmol/L) with ketone levels >1.5 mmol/L. Conversely, when interstitial glucose is >270 mg/dL (>15.0 mmol/L), trending upward, and ketone levels are <1.5 mmol/L, the app provides short continuous aerobic exercise sessions to support insulin bolus–mediated correction of hyperglycemia before reassessing eligibility for resistance training. As a further update, Diactive-1 (v.2.0) includes “Did you know?” loading-screen messages summarizing findings from scientific studies linking muscular strength with health benefits, aiming to make research evidence accessible to youth with type 1 diabetes.

Diactive-1 (v2.0) incorporates two gamification components designed to support user engagement in health-related behavior change. First, a point- and level-based progression system awards XP for completed weekly sessions, with additional XP for sustained streaks. Consecutive training days are displayed as flame icons, and accumulated XP allows participants to progress through user levels and appear in a nickname-coded ranking stratified by biological sex and age group. These level-feedback (31), and streak-based systems (32), are recognized gamification strategies used in health and physical activity mHealth interventions. Second, an achievement system unlocks 80 badges classified as common, rare, or epic. This system corresponds to the achievement attribute of gamification, which has been identified as a relevant motivational component in healthcare contexts (33). To further enhance usability and engagement, Diactive-1 (v.2.0) can be configured in either Spanish or English. In addition, participants may link their Spotify profile to the app, allowing them to listen to their own music during training sessions. These features are intended to support accessibility, personalization, and user experience during the intervention.

**Figure 3:**
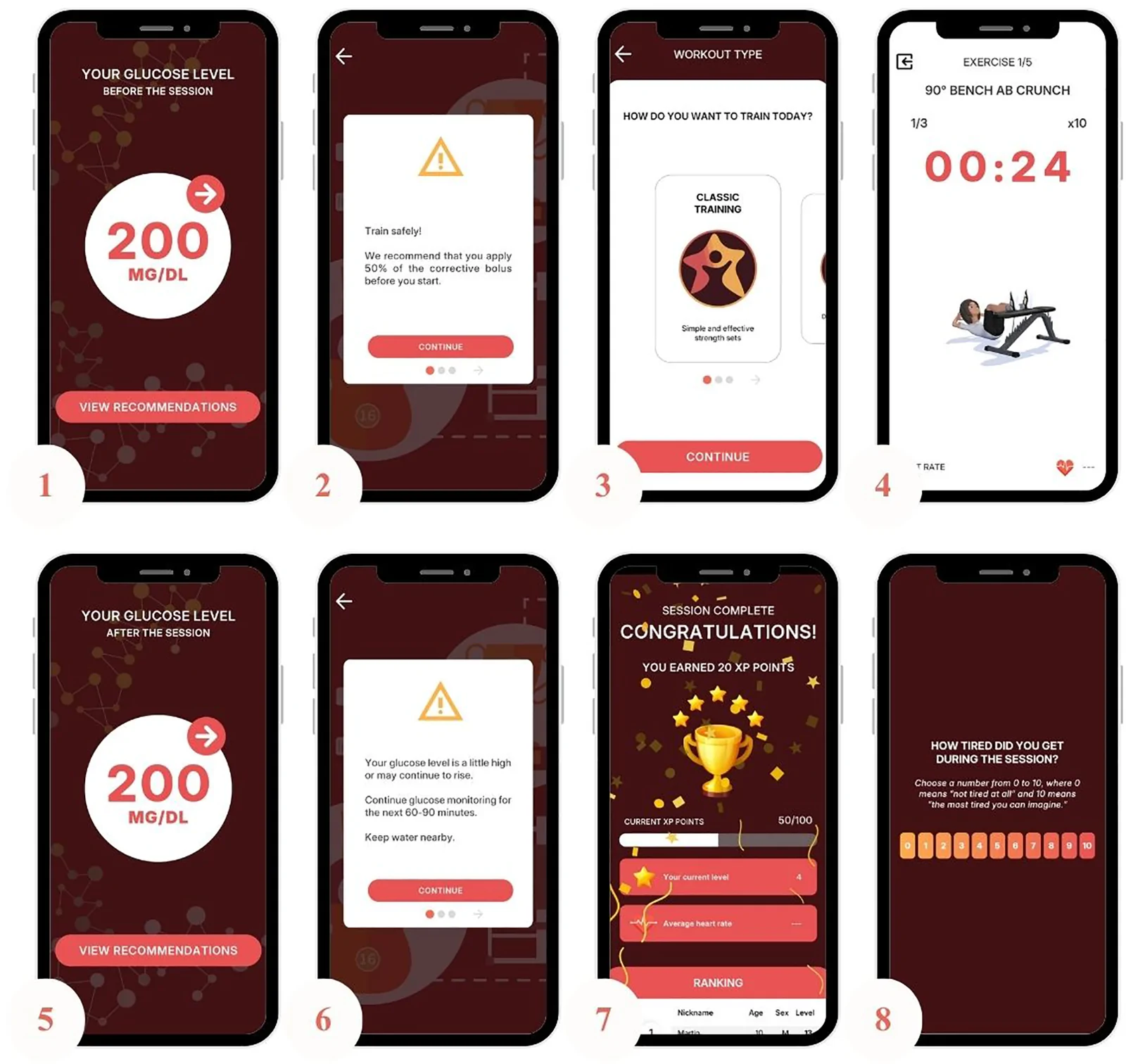
Sequence of the Diactive-1 training session, including pre-exercise glucose assessment, safety recommendations, exercise selection and execution, post-exercise glucose assessment, gamification, and perceived exertion reporting.

During all sessions, a randomly assigned 3D avatar (i.e., either a boy or a girl) guides participants by demonstrating the exercises. The avatar is accompanied by narrated instructions, including technique cues, transitions for unilateral exercises, and rest-period duration, to support safe training. **Figure 3** illustrates the sequence of a Diactive-1 (v2.0) training session.

#### 2.8.4 Intervention participation measurement

Intervention participation will be assessed through app access data, and completed sessions will be defined as sessions that are started and fully completed; the type of session, the number of exercises performed, and the number of sets and repetitions will be recorded. At the end of each session, participants will report their perceived exertion using the modified Borg scale from 0 (“not intense”) to 10 (“extremely intense”) (34). Thus, participation data will capture both session completion and the perceived intensity of each completed session for all participants. Every Thursday, participants will receive a notification indicating the number of sessions completed that week, allowing three remaining days within the Monday-to-Sunday period to meet ADA recommendations (20).

These measures will be supplemented by mean and maximum heart rate recorded using the Polar Verity Sense monitor (Polar Electro, Kempele, Finland) in participants recruited from four randomly selected hospitals. Because of resource constraints, heart-rate monitoring will be limited to this subsample and will provide complementary objective information rather than define intervention exposure. Exercise execution quality will not be formally assessed; however, standardized tutorials, 3D exercise demonstrations, and narrated technique cues will be provided to support correct and safe execution.

### 2.9 Primary outcome

The primary outcome will be the change in age- and sex-standardized handgrip strength z-score from baseline to 12 weeks. Handgrip strength was selected as the primary outcome because it is a feasible, standardized, and clinically practical indicator of muscular fitness that can be assessed consistently across participating hospitals and showed a significant between-group improvement in the previous single-center Diactive-1 trial (10). The trial will assess whether participants allocated to the intervention group show greater improvement in handgrip strength z-score than those allocated to the usual care group. Handgrip strength will be measured with the Takei III Smedley Type 297 digital dynamometer (Takei Scientific Instruments, Niigata, Japan), following The Youth Fitness International Test (YFIT) (35). Each participant will perform two maximal trials alternately with each arm, and the best value for each hand will be recorded. The average of the best right- and left-hand values will be used as the absolute handgrip strength value. Handgrip strength will then be standardized using FitBack^©^ sex- and age-specific reference values to obtain z-scores (30). Absolute handgrip strength in kilograms will be reported as a secondary outcome. This approach is supported by preliminary Diactive-1 trial findings showing a significant between-group improvement in handgrip strength z-score at 12 weeks (23).

### 2.10 Secondary outcomes

Secondary outcomes assessed in Diactive-1 are summarized in **Table 1**. Secondary outcomes are organized hierarchically in: key secondary outcomes (i.e., glycemic-stability parameters and insulin requirements); descriptive or contextual outcomes (i.e., sociodemographic information, diabetes-related clinical characteristics, anthropometric parameters, pubertal maturation, intervention engagement, and barriers to physical activity); and exploratory or hypothesis-generating outcomes, including cardiovascular biomarkers and lipid profile; lifestyle-related outcomes (i.e., 24-hour movement behaviors and adherence to the Mediterranean diet); physical-domain outcomes (i.e., self-reported physical fitness and physical literacy); mental and psychosocial outcomes (i.e., psychological assessments, health-related quality of life, and screening for eating disorders); and implementation-related outcomes intended to explore usability, acute glycemic responses, and potential dose-response relationships (i.e., app usability and data recorded by the Diactive-1 app).

**Table 1:** Summary of the variables examined in the randomized clinical trial.

Primary outcome	Measurement	Tool
Handgrip strength	Directly: Objectively	- Takei III Smedley Type 297 digital dynamometer. - Age- and sex-standardized z-score calculated using FitBack^©^ reference values.
Secondary Outcome	Measurement	Tool
Glycemic stability	Indirectly: - Objectively	- CGM downloads. (without brand distinction)
Insulin requirements	Indirectly: - Objectively - Self-reported	- Insulin pump downloads. - Smart-pen logs. - Insulin records from participants using CGM. (without brand distinction)
Cardiovascular biomarkers and lipid profile	Directly: - Objectively	- Laboratory testing at each hospital’s central laboratory. (standardized protocols)
Anthropometric parameters	Directly: - Objectively	- Height: SECA 213 stadiometer. - Weight: SECA electronic scale.
Maturity status	Indirectly: - Objectively	- Peak height velocity: Moore’s equation. - Tanner scale.
Self-reported fitness	Subjectively: - Self-reported	- International Fitness Scale (IFIS)
Physical literacy	Subjectively: - Self-reported	- Spanish Perceived Physical Literacy Instrument (S-PPLI)
24-Hour Movement Patterns	Subjectively: - Self-reported	- Physical activity: Physical Activity Questionnaire for Children (PAQ-C), or Physical Activity Questionnaire for Adolescents (PAQ-A). - Screen time: selected items from the Health Behavior in School-aged Children (HBSC) questionnaire. - Sleep: selected items from the Pittsburgh Sleep Quality Index (PSQI).
Barriers to physical activity	Subjectively: - Self-reported	- Physical Activity Barriers Scale-1 (PABS-1).
Sociodemographic information (baseline only)	Subjectively: - Self-reported	- Family Affluence Scale III (FAS III)
Psychological assessments	Subjectively: - Self-reported	- CUBE questionnaire.
Quality of life for youth	Subjectively: - Self-reported	- DISABKIDS questionnaire. - KIDSCREEN-10 Index.
Quality of life for families	Subjectively: - Self-reported	- DISABKIDS questionnaire for parents. - KIDSCREEN-10 Index for parents.
Mediterranean diet	Subjectively: - Self-reported	- Mediterranean Diet Quality Index for Children and Adolescents (KIDMED).
Eating disorders	Subjectively: - Self-reported	- Sick, Control, One, Fat, Food questionnaire (SCOFF)
App usability users	Subjectively: - Self-reported	- User Version of the Mobile Application Rating Scale (uMARS).
App usability experts	- Expert-rated	- Mobile Application Rating Scale (MARS).
Diactive-1 app parameters	Directly: - app-recorded	Diactive-1 (v.2.0) app: - Session date and time. - Training type. - Pre- and post-session glucose levels and trend arrows. - Hypoglycemia events. - Mean and maximum heart rate, when available in the heart-rate monitoring subsample. - Satisfaction and perceived exertion levels. - Number of exercises, sets, and repetitions. - Recovery times.

#### 2.10.1 Glycemic stability

Glycemic parameters, including CGM wear time, mean glucose, glucose management indicator (GMI), time well above range (%TwAR; >250 mg/dL [>13.9 mmol/L]), time above range (%TAR; 180–250 mg/dL [10.1–13.9 mmol/L]), time in range (%TIR; 70–180 mg/dL [3.9–10.0 mmol/L]), time below range (%TBR; 54–70 mg/dL [3.0–3.8 mmol/L]), time well below range (%TwBR; <54 mg/dL [<3.0 mmol/L]), glycemic coefficient of variation (CV), hypoglycemic events (i.e., values <70 mg/dL [<3.9 mmol/L] lasting ≥15 min), and nocturnal hypoglycemic events (i.e., hypoglycemic events between 10:00 p.m. and 08:00 a.m.), will be obtained from CGM software regardless of the brand used.

Data will be collected during continuous 14-day monitoring periods before the baseline and 12-week medical visits (36). For descriptive and exploratory purposes, CGM data will also be collected during the 7 days after each visit, corresponding to the period in which carbohydrate intake and insulin-correction doses are prospectively recorded (37).

Glycemic targets will be evaluated according to ADA guidelines (38): HbA1c <7%, CV ≤36%, TwAR <5%, TAR <25%, TIR >70%, TBR <4%, and TwBR <1%. The glycemic risk index (GRI) score will be calculated as follows: (3.0 × TwBR) + (2.4 × TBR) + (1.6 × TwAR) + (0.8 × TAR) (39).

#### 2.10.2 Insulin requirements

During pre-project meetings, clinical teams noted that young people not using AID pumps do not routinely enter insulin data into their CGM systems. Therefore, at the baseline and 12-week visits, these participants will be instructed to prospectively record carbohydrate intake and insulin doses for the following 7 days. Daily carbohydrate intake and insulin doses, including basal insulin, rapid-acting boluses, correction boluses, and total daily insulin, will be collected and averaged over this 7-day period.

For participants using AID pumps, carbohydrate intake and insulin-dose data will be extracted from the corresponding pump software, regardless of device brand. Data will be obtained for the 7 days following each visit to ensure comparability with participants not using AID pumps. In addition, 14-day pre-visit data will be extracted for AID pump users and used in future within-group subanalyses.

#### 2.10.3 Diabetes-related clinical characteristics and complications

Diabetes-related clinical characteristics will be collected from clinical records and the Diactive-1 web-based platform at baseline and, when applicable, at the 12-week assessment. These variables will include date of type 1 diabetes onset, diabetes duration, insulin delivery modality, use of multiple daily injections or insulin pump therapy, type of insulin pump, use of automated insulin delivery systems, CGM device type, type of rapid-acting insulin, type of long-acting insulin when applicable, prescribed carbohydrate intake, and current diabetes-related treatment characteristics.

Available information on diabetes-related complications will also be recorded from clinical records, including retinography results, nephropathy, or microalbuminuria status when available, and other clinically documented microvascular complications. These variables will be used mainly for baseline characterization, descriptive analyses, and exploratory subgroup analyses.

#### 2.10.4 Cardiovascular biomarkers and lipid profile

Fasting blood and urine samples will be collected in the morning after an overnight fast of at least 12 h at the baseline and 12-week assessments, according to routine clinical procedures at each participating hospital. Samples will be processed in the corresponding hospital laboratory by technicians not involved in the project.

Core biochemical variables will include HbA1c (% and mmol/mol), serum glucose (mg/dL), lipid profile markers, renal-function markers, and urine albumin-related biomarkers. Specifically, when available, blood analyses may include urea and creatinine (mg/dL), total cholesterol, HDL-C, calculated LDL-C and triglycerides (mg/dL), ApoA-1 and ApoB-100 (mg/dL), albumin (g/L), liver enzymes (U/L), sodium and potassium (mmol/L), calcium (mg/dL), cystatin C (mg/L), and 25-hydroxyvitamin D (ng/mL). Urine analyses may include urinary microalbumin or albumin concentration, urinary creatinine, albumin-to-creatinine ratio when available, and routine urine-strip parameters. Complete blood count parameters will also be collected when available.

Given the multicenter pragmatic design, not all biomarkers may be available across all participating hospitals. Therefore, biomarkers will be analyzed according to availability and harmonized units, with core variables prioritized for between-group analyses and additional variables used for exploratory or descriptive analyses. Estimated glomerular filtration rate will be calculated using the bedside Schwartz equation, when serum creatinine is available, and expressed as mL/min/1.73 m² (40).

#### 2.10.5 Anthropometric parameters and stage of maturity

Standing height will be measured to the nearest 0.1 cm using a SECA 213 stadiometer, with participants barefoot, heels together against the base of the vertical column, back straight, and head positioned in the Frankfurt horizontal plane (41). Body weight will be measured to the nearest 0.1 kg using a SECA 869 electronic scale, with participants barefoot and wearing light clothing. BMI will be calculated as weight in kilograms divided by height in meters squared (kg/m^2^).

Peak height velocity (PHV) will be estimated using the Moore equation (42), and categorized as prepubertal (≤−1 year from PHV), peri-pubertal (−1 to 1 year from PHV), or post-pubertal (≥1 year from PHV) (43). Pubertal status will be assessed using Tanner criteria (44), which will be examiner-rated (i.e., physician-assessed) during the clinical visit.

#### 2.10.6 Self-reported physical fitness

Self-reported physical fitness will be assessed using the International Fitness Scale (IFIS), a validated 5-item questionnaire evaluating perceived overall physical fitness, cardiorespiratory fitness, muscular fitness, speed–agility, and flexibility. The IFIS has shown good test–retest reliability (45). Each item is scored from 1 (“very poor”) to 5 (“very good”), with higher scores indicating better perceived fitness. The overall fitness item will be used as the global indicator, while the remaining items will describe specific fitness domains. If required, a composite IFIS score will be calculated by summing all five items, ranging from 5 to 25 points.

#### 2.10.7 Physical literacy

Perceived physical literacy will be assessed using the Spanish Perceived Physical Literacy Instrument for Adolescents (S-PPLI), a culturally adapted and validated version of the Perceived Physical Literacy Instrument for use in Spanish adolescents. The S-PPLI has shown adequate construct validity and reliability (46).

The S-PPLI comprises 9 items across three 3-item factors. Each item is scored on a 5-point Likert scale from 1 (“strongly disagree”) to 5 (“strongly agree”). Factor scores are obtained by summing their three items, ranging from 3 to 15 points. The global S-PPLI score is the sum of all nine items, or the three factor scores, ranging from 9 to 45 points, with higher scores indicating greater perceived physical literacy.

The global S-PPLI score will be analyzed as a continuous variable, with factor-specific scores used to describe the three perceived physical literacy components. When categorical interpretation is needed, previously used cut-offs will classify participants as low (9–31), moderate (32–36), or high (37–45) perceived physical literacy.

#### 2.10.8 Twenty-four-hour movement behaviors

*Physical activity:* Self-reported physical activity will be assessed using the Spanish versions of the Physical Activity Questionnaire for Children (PAQ-C) (47), and the PAQ for Adolescents (PAQ-A) (48), according to participants’ age. Children aged 8–12 years will complete the PAQ-C, while adolescents aged 13–17 years will complete the PAQ-A. Both questionnaires are self-administered 7-day recall instruments designed to estimate general moderate-to-vigorous physical activity. The Spanish PAQ versions have shown good test–retest reliability and reasonable criterion validity against accelerometer-derived physical activity.

The PAQ-C comprises 10 items, nine of which contribute to the global score and assesses leisure-time activity, physical education, recess, lunchtime, after-school and evening activity, weekend activity, overall weekly activity, and daily activity frequency. The PAQ-A comprises 9 items, eight of which are scored, and assesses the same domains except recess, which is omitted. In both questionnaires the final item identifies illness and is not scored.

Scored responses will be coded from 1 to 5, with higher values indicating higher physical activity. The leisure-time domain will be computed as the mean of the listed activities, and the daily-frequency domain as the mean of the seven weekday scores. Original PAQ scores will be calculated as the mean of all scored domains: nine for the PAQ-C and eight for the PAQ-A, as the adolescent version omits the recess domain. To harmonize both questionnaires, a common PAQ score will be derived from the eight shared domains.

*Screen time:* Screen time will be assessed using selected items from the Spanish version of the Health Behavior in School-aged Children (HBSC) questionnaire, a WHO collaborative cross-national study that uses standardized school-based self-report questionnaires (49). Participants will report their usual leisure-time screen use separately for weekdays and weekend days across three domains: television/videos, electronic games, and other electronic-device use. For each domain, average daily screen time will be calculated as a weighted mean of weekday and weekend use: [(weekday use × 5) + (weekend use × 2)]/7. Domain-specific estimates will then be summed to derive total leisure-time screen time. The HBSC screen-time items have shown acceptable test–retest reliability in adolescents, supporting their use for population-based surveillance of screen-based behaviors (50).

*Sleep time and efficiency:* Sleep will be assessed using selected items from the Pittsburgh Sleep Quality Index (PSQI) (51). Participants will report their usual bedtime, wake-up time, sleep-onset latency, and actual sleep duration during the past month. Sleep efficiency will be estimated as actual sleep duration divided by time in bed, multiplied by 100.

#### 2.10.9 Barriers to physical activity

Perceived barriers to physical activity will be assessed using the Physical Activity Barriers Scale for pediatric type 1 diabetes (PABS-1), an 18-item self-administered questionnaire developed and validated in Spanish-speaking children and adolescents with type 1 diabetes (52). The PABS-1 has shown adequate psychometric properties in youth with type 1 diabetes aged 6–18 years. Content validity was supported by experts and the four-factor structure showed factorial validity. Test–retest reliability showed moderate-to-good stability for most items.

Items are scored on a 4-point Likert scale from 0 (“strongly disagree”) to 3 (“strongly agree”), with higher scores indicating greater perceived barriers. Total and domain-specific mean scores will be calculated across four domains: diabetes-related barriers, physical and health-related barriers, motivational barriers, and environmental and family-related barriers.

#### 2.10.10 Sociodemographic information

Variables will include date of birth, age, biological sex, and date of onset of diabetes. Participant identifiers such as nickname or contact email will be recorded exclusively for study management and access to the intervention platform and will not be used as analytical variables.

Socioeconomic status will be estimated using the Family Affluence Scale III (FAS-III) (53). The FAS-III comprises six items assessing: car ownership, 0–2 points; individual bedroom, 0–1 point; number of computers/tablets, 0–3 points; number of bathrooms, 0–3 points; dishwasher ownership, 0–1 point; and family vacations abroad, 0–3 points. The total FAS-III score is obtained by summing all item scores, ranging from 0 to 13 points, with higher values indicating greater family affluence. Participants will be categorized as having low (0–2 points), medium (3–5 points), or high (≥6 points) status.

#### 2.10.11 Psychological assessments

Subjective well-being will be assessed using the *Cuestionario Único de Bienestar Escolar* (CUBE), a validated questionnaire designed to evaluate cognitive and affective components of well-being in children and adolescents (54). The instrument comprises three domains: life satisfaction, positive emotions, and negative emotions. Life satisfaction is assessed through five items scored on a 10-point Likert scale ranging from 0 (“totally disagree”) to 10 (“totally agree”). The emotional items are described as using a 5-point Likert scale ranging from 0 to 5, which comprises six response values.

Negative emotion scores will be reverse-coded so that higher values consistently indicate greater well-being. Because life satisfaction and emotional domains are measured using different response scales, all domain scores will be rescaled to improve interpretability. Domain-specific scores and an overall subjective well-being score will then be calculated, with higher scores indicating better subjective well-being. This questionnaire has previously been used in the Diactive-1 cohort to assess subjective well-being in children and adolescents (55).

#### 2.10.12 Generic health-related quality of life

Generic health-related quality of life will be assessed using the KIDSCREEN-10, a validated 10-item unidimensional questionnaire for children and adolescents aged 8–18 years (56). Both the youth self-report and parent-proxy versions will be administered. The KIDSCREEN-10 assesses global perceived health-related quality of life over the previous week, covering physical well-being, energy, mood, loneliness, autonomy and leisure time, family relationships, peer relationships, school functioning, and attention at school. Items are rated on a 5-point Likert scale, ranging from “never” to “always” or from “not at all” to “extremely,” depending on the item. After reverse-coding negatively worded items so that higher scores indicate better health-related quality of life, the KIDSCREEN-10 items will be summed to obtain a raw score, which will be converted into the corresponding Rasch-person parameter and transformed into a norm-based T-score according to the official scoring procedure.

#### 2.10.13 Diabetes-specific health-related quality of life

Diabetes-specific health-related quality of life will be assessed using the Spanish version of the DISABKIDS diabetes-specific module (57). Both the youth self-report and parent-proxy versions will be administered to capture the participant’s own and the parent’s or guardian’s proxy perception. The module comprises 10 diabetes-specific items organized into two dimensions reflecting the impact of the disease and the impact of treatment on health-related quality of life. Items are answered on a 5-point Likert scale ranging from 1 (“never”) to 5 (“always”). As all items indicate diabetes-related burden, responses will be reverse-transformed to a 0–100 scale so that higher scores indicate better diabetes-specific health-related quality of life. If an overall diabetes-specific health-related quality of life score is required, it will be derived by combining both dimensional scores as an item-weighted mean according to the number of items contributing to each dimension.

#### 2.10.14 Adherence to Mediterranean diet

To evaluate adherence to the Mediterranean diet, the Mediterranean Diet Quality Index for Children and Teenagers (KIDMED) index will be applied (58). This test was previously validated (58), and widely used in the Spanish young population with diabetes (59). The KIDMED index ranges from 0 to 12 and is based on a 16-question test. Items reporting unhealthy characteristics are scored with −1 point, and those reporting healthy characteristics with +1 point. The sum of scores will be used to categorize into 3 levels: (a) optimal Mediterranean diet (>8 points), (b) improvement needed (4 to 7 points), (c) low diet quality (≤3 points).

#### 2.10.15 Screening for eating disorders

The risk of having eating disorders will be assessed by the Sick, Control, One, Fat, Food (SCOFF) questionnaire, a five-question test that can be self-administered with an acceptable sensitivity and specificity (60). The Spanish SCOFF questionnaire version has been validated for its use in primary care settings (60). A score ≥2 will indicate risk of eating disorders.

#### 2.10.16 App usability

*Expert evaluation:* The Spanish adaptation of the Mobile Application Rating Scale (MARS) will be used for standardized expert assessment of mHealth app quality (61). The MARS includes 23 items rated on a 5-point Likert scale from 1 (“inadequate”) to 5 (“excellent”), organized into four objective quality domains (engagement, functionality, aesthetics, and information quality) and one subjective quality domain. Each domain score will be calculated as the mean of its corresponding items. The overall app quality score will be computed as the mean of the four objective domain scores, while the subjective quality score will be reported separately.

*User evaluation:* User-perceived app quality and usability will be assessed using the Spanish version of the User Version of the Mobile Application Rating Scale (uMARS). The uMARS comprises 20 items rated on a 5-point Likert scale from 1 (“inadequate”) to 5 (“excellent”) and is organized into four domains (engagement, functionality, aesthetics, and information quality). Domain scores will be calculated as the mean of their corresponding items, and the overall app quality score will be computed as the mean of the four domain scores. To complement the uMARS assessment, three open-ended questions will be included to explore users’ perceptions of the app, specifically the most valued features, difficulties encountered during use, and suggestions for improvement. These qualitative responses will provide contextual information.

#### 2.10.17 Data recorded by the Diactive-1 app

Intervention delivery and participation data will be captured through the Diactive-1 app throughout the study period. For each completed session, recorded variables will include the session number, date and time of completion, time since training onset, training type, specific exercises performed, number of exercises, sets and repetitions, activity times, and recovery times. The app will also record diabetes management variables, including pre- and post-session glucose values, pre- and post-session glucose trend arrows, hypoglycemic events reported since the previous session, heart rate metrics when available, and subjective perception of exertion after the session. User-reported session satisfaction will also be collected.

### 2.11 Participant timeline

Each participant will follow a standardized timeline comprising screening and enrolment, baseline assessment, randomization, a 12-week intervention or usual-care period, post-intervention assessment, and post-T1 data collection. Screening and informed consent or assent may be initiated before the baseline visit.

Baseline procedures will be completed in the following sequence: (i) a baseline clinical visit, during which eligibility will be confirmed, informed consent or written assent will be verified, and sociodemographic, diabetes-related clinical, anthropometric, pubertal-maturation, and handgrip-strength data will be collected; (ii) central randomization immediately after the baseline clinical data have been entered into the Diactive-1 web platform; (iii) completion of the online web-based questionnaires within 48 hours after the baseline visit; (iv) a 7-day baseline data-collection period, during which participants will undergo fasting blood and urine testing and upload all available glucose-monitoring, insulin, and carbohydrate-intake data from their CGM system or insulin pump; (v) upload of the laboratory results and CGM or insulin-pump reports by the clinical research team within the following 72 hours, including glucose-monitoring data covering the 14 days before and 7 days after the baseline clinical visit; and (vi) verification of the completeness of all baseline information, followed by activation of the Diactive-1 app for participants allocated to the intervention group. Thus, intervention exposure will begin only after all baseline assessments and data-collection procedures have been completed and verified. The 12-week intervention or usual-care follow-up period will begin on the date of app activation or the corresponding date for participants allocated to usual care. To facilitate scheduling of the final clinical assessment, the web platform will generate automated alerts 15 and 7 days before the expected Week 12 assessment.

**Figure 4:**
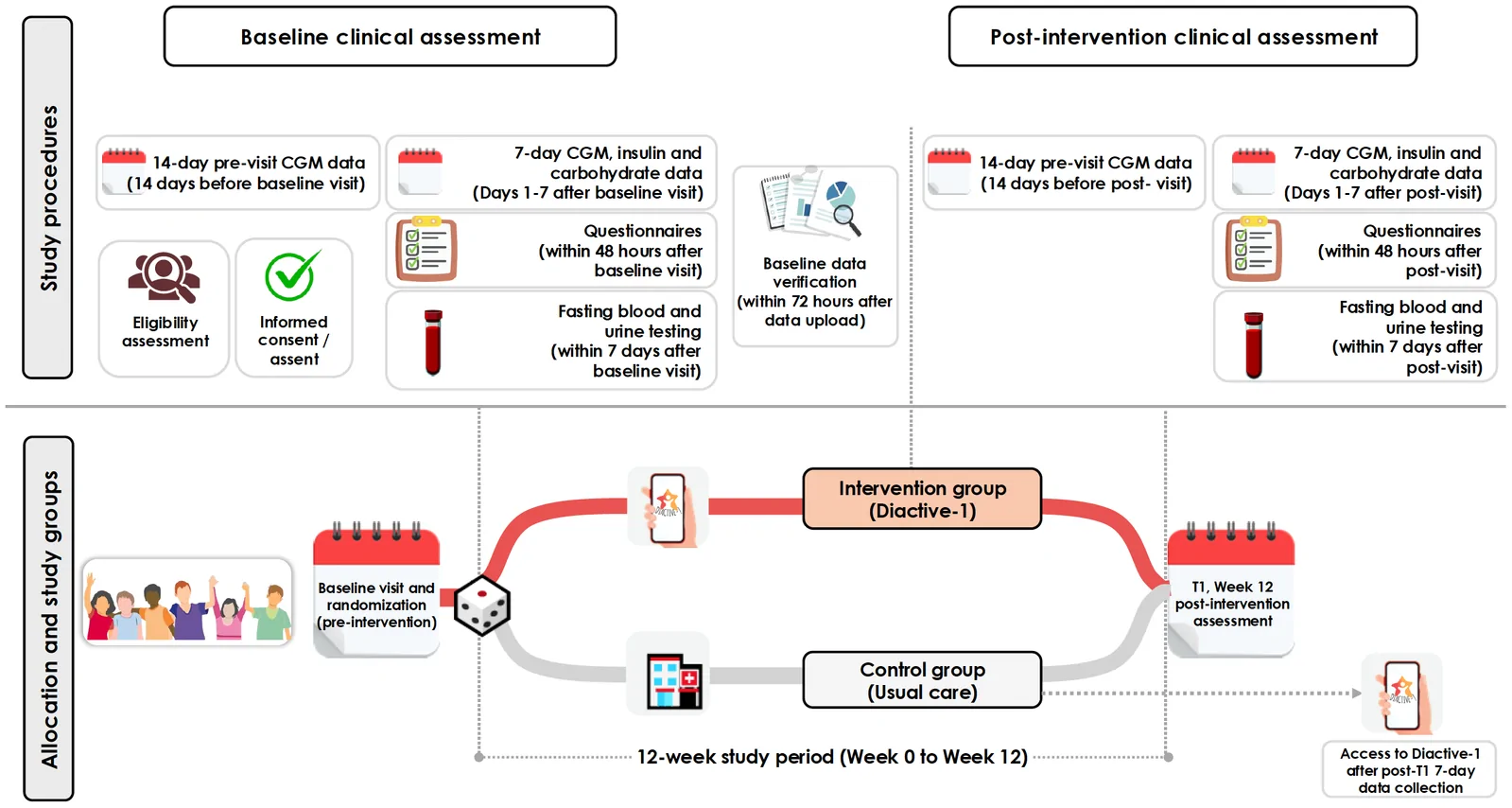
Overview of the Diactive-1 study timeline, including baseline procedures, randomization, intervention and usual-care periods, and post-intervention assessments.

At Week 12, measured from the beginning of the intervention or usual-care follow-up period, the post-intervention procedures will replicate the baseline assessment protocol. Once these data have been uploaded, checked, and verified as complete, participants in the Diactive-1 group will be considered to have completed the intervention but will retain access to the app for continued voluntary use, whereas participants in the usual-care control group will be granted access to the Diactive-1 mHealth app. **Figure 4** provides an overview of the project timeline, and **Table 2** presents the schedule of enrolment, interventions, and assessments.

**Table 2:** Schedule depicting the enrollment and interventions for the Diactive-1 Study in accordance with the SPIRIT 2025 guidelines.

Randomization	TRIAL PERIOD			
	Enrollment		Pre-randomization	Post-randomization
Timepoint	*-t_i_* to 0	0	*Baseline t_0_*	*t_1 (12 weeks)_*
ENROLLMENT:				
Eligibility screen	X	X*		
Informed consent	X	X*		
Randomization		X		
INTERVENTION/COMPARATOR:				
Diactive-1 ^a^			↔	↔
Usual care ^b^			↔	↔
ASSESSMENTS:				
Handgrip strength			X	X
Glycemic stability			X	X
Insulin requirements			X	X
Cardiovascular biomarkers and lipid profile			X	X
Anthropometric parameters			X	X
Maturity status			X	X
Self-reported fitness			X	X
Physical literacy			X	X
24-Hour Movement Patterns			X	X
Barriers to physical activity			X	X
Sociodemographic information			X	
Psychological assessments			X	X
Quality of life for youth			X	X
Quality of life for families			X	X
Mediterranean diet			X	X
Eating disorders			X	X
App usability (users/experts)^c^				X
Diactive-1 app parameters^d^			↔	↔

Note: X indicates an assessment or procedure performed at the corresponding timepoint; ↔ indicates continuous delivery, usual-care exposure, or app-based data recording throughout the 12-week study period. Baseline clinical assessments will be completed before randomization, whereas the remaining baseline questionnaires and 7-day diabetes-related data collection will be completed after randomization but before intervention activation.

*Eligibility and consent may be screened before enrolment and confirmed at the baseline visit before randomization.

^a^ Diactive-1 will be activated only after all baseline procedures and data have been completed and verified and will then be delivered for 12 weeks.

^b^ Participants allocated to the usual-care/waiting-list control group will receive usual clinical care during the 12-week study period and will be offered Diactive-1 access after post-T1 7-day data collection.

^c^ App usability will be assessed at the end of the intervention period among participants with access to the app; expert MARS assessment will be reported separately if not linked to participant follow-up.

^d^ Diactive-1 app parameters will be recorded continuously during app use and summarized at the end of the intervention period.

### 2.12 Adverse event reporting and harms

At all participating hospitals, usual care includes routine pediatric diabetes follow-up, review of glucose and insulin data, diabetes self-management counseling, and treatment adjustment when clinically indicated. Routine care will not be standardized across sites. Each hospital will provide a brief description of its exercise counseling, follow-up practices, and routine use of CGM, insulin pumps, and automated insulin-delivery systems. Unscheduled clinical contacts and any clinically relevant changes in treatment or diabetes technology during follow-up will be recorded. Adverse events and other health problems will be documented separately from these treatment-related changes.

Moreover, adverse events will be actively monitored at the scheduled study visits and throughout the intervention through reports from participants or their guardians and through contacts with the clinical-research team. Participants and guardians will be instructed to report any health problems occurring during the study, irrespective of its presumed relationship with the intervention. All reported health problems and adverse events will also be recorded as incidents. In addition, each time participants open the Diactive-1 app after a completed session, they will be asked whether they experienced any hypoglycemic episode in the hours following the previous Diactive-1 session. These app-recorded post-session hypoglycemic events will be used to complement adverse-event monitoring and will be reviewed by the clinical-research team when appropriate. All adverse events will be recorded in the case report form.

Exercise sessions may be temporarily interrupted or permanently discontinued in the event of a serious adverse event, recurrent severe hypoglycemia, persistent hyperglycemia with ketosis, acute illness, injury, or any new medical condition contraindicating exercise participation. Discontinuation of the intervention for these motives will not automatically imply withdrawal from study follow-up, unless requested by the participant or guardian.

Serious adverse events will be reported to the relevant ethics committee and competent institutional bodies according to local regulations and hospital-specific procedures.

### 2.13 Data collection methods and management

Study data will be collected using standardized electronic case report forms and stored in a secure, pseudonymized database. Identifying information will be stored separately from research data. Direct identifiers, such as name and contact information, will only be accessible to authorized healthcare professionals at the recruiting site, or to authorized coordinating personnel when strictly required for study management or participant safety. The coordination team will routinely access pseudonymized study data only.

Data will be hosted on an encrypted Google Cloud Platform server using Cloud Firestore, a scalable NoSQL database, in the Europe-west region (Zurich, Switzerland). Access to the platform and database will be role-based, password-protected, and restricted to the minimum information required for each user profile. Any data correction after verification will require an authorized and documented amendment, with an audit trail maintained within the system.

Data handling, storage, and protection procedures will comply with Regulation (EU) 2016/679, the General Data Protection Regulation, and Spanish Organic Law 3/2018 on Personal Data Protection and guarantee of digital rights.

### 2.14 Data monitoring

Data and safety monitoring will be performed by the principal investigator and coordinating center throughout the trial. Monitoring will include remote review of recruitment and follow-up status, informed consent documentation, data completeness and consistency, adverse event records, app-recorded safety information, and adherence to intervention and assessment procedures. Deviations or data-quality issues will trigger documented corrective actions. The Diactive-1 web portal will provide a timestamped audit trail of relevant user actions, enabling remote verification of data integrity and site compliance. No formal data monitoring committee will be established, as the intervention is low-risk, non-pharmacological, and conducted alongside standard clinical care. Safety monitoring will remain independent of the funder, and any potential conflicts of interest will be disclosed in the “Funding” and “Conflicts of Interest” sections.

No interim efficacy analyses or formal stopping rules have been planned. However, if safety concerns, recurrent serious adverse events, or protocol-related risks are identified, the principal investigator may recommend temporary suspension, protocol amendment, or early termination of the trial. Any such decision will be documented and reported to the relevant ethics committee and institutional bodies in accordance with applicable regulations and local procedures.

### 2.15 Statistical analysis

The R version and packages used will be reported. Cross-sectional analyses with baseline data will follow the STROBE statement, while the final reporting of the RCT will adhere to CONSORT 2025 (62). A two-sided α level of 0.05 will be used to determine statistical significance for the primary outcome.

Normality, descriptive statistics and group differences: Distributional properties of continuous variables will be evaluated through graphical inspection (i.e., histograms and Q–Q plots) and formally assessed using the Shapiro–Wilk test when appropriate. Homogeneity of variance will be examined using Levene’s test. Continuous variables will be summarized as mean ± standard deviation (SD) when approximately normally distributed, or as median and interquartile range (IQR) otherwise. Categorical variables will be presented as absolute and relative frequencies. Baseline characteristics will be summarized by randomized group using descriptive statistics, without formal hypothesis testing. Any baseline differences will be interpreted descriptively and clinically.

Missing data: Missing data will be examined using graphical and tabular summaries by variable, treatment group, and time point. The number and percentage of missing observations and missingness patterns will be reported separately by randomized group. Compatibility with a missing completely at random (MCAR) mechanism will be assessed using Little’s MCAR test, together with comparisons of baseline characteristics between participants with and without missing outcome data as a supplementary analysis. A missing-at-random (MAR) mechanism will be explored by modeling missingness indicators as a function of observed variables. A missing-not-at-random (MNAR) mechanism will be considered when missingness remains plausibly related to unobserved values or post-randomization events not fully explained by observed data.

The primary intention-to-treat (ITT) analysis will use all available outcome data through maximum-likelihood estimation in the prespecified linear mixed-effects model under a MAR assumption. This likelihood-based approach does not require explicit imputation of missing outcomes and is appropriate for longitudinal continuous outcomes when at least MAR is considered plausible (63). Multiple-imputation sensitivity analyses will be conducted if the primary outcome at 12 weeks is missing for at least 5% of the participants randomized to either treatment group (64). Multiple imputation will also be performed if the reasons for missingness raise concerns about the plausibility of the MCAR or MAR assumption. Then, multiple imputations will be performed using chained equations within each randomized group (65), including baseline and post-intervention measurements, hospital, and potential variables associated with missing data for the primary outcome.

Cross-sectional analysis: Associations between categorical variables will be examined using Pearson’s chi-square test or Fisher’s exact test, as appropriate. For quantitative variables, associations will be assessed using Pearson’s correlation coefficient or Spearman’s rho (ρ), depending on distributional assumptions and the linearity of the association. Between-group comparisons, when relevant for cross-sectional exploratory analyses, will be performed using independent-samples t tests or one-way analysis of variance for approximately normally distributed variables, and Mann–Whitney U or Kruskal–Wallis tests for non-normally distributed variables. Regression analyses will be performed using the modeling framework most appropriate to the scale and distribution of the outcome, the functional form of the association, and the data structure. Model selection will therefore consider normality, homoscedasticity, linearity, and clustering or nesting when applicable.

Effect of the intervention: The intervention effect will be estimated for the primary outcome using linear mixed-effects models under the ITT principle, with participants analyzed according to randomized allocation. Baseline and 12-week outcome values will be jointly modeled as repeated outcome measurements using data in long format. Models will include fixed effects for group, time, and the group × time interaction, with random intercepts for hospital and participant nested within hospital. Because baseline outcome values are included as repeated measurements, they will not be entered as separate covariates. The primary treatment effect will be estimated from the group × time interaction, representing the between-group difference in change from baseline to 12 weeks (i.e., over time). The same modeling framework will be applied to the prespecified secondary continuous outcomes, with correction for multiple testing within outcome families using the Benjamini–Hochberg false discovery rate procedure (66).

The primary model for handgrip strength will include no additional covariates. Prespecified adjusted models for glycemic outcomes will additionally include baseline daily insulin dose and baseline carbohydrate intake, whereas models for daily insulin dose will include baseline carbohydrate intake. These covariates were selected *a priori* because of their established prognostic relationships with the corresponding outcomes. No other covariates will be included in these prespecified models (67). Any additional unplanned adjusted analyses will be clearly identified as *post hoc* sensitivity analyses and will not be selected solely on the basis of observed baseline differences between groups (62).

In addition to the primary ITT analysis, per-protocol (PP) analyses will be conducted including intervention participants who complete an average of at least two resistance-training sessions per week during the 12-week intervention (68), provided that these sessions are reasonably distributed across the intervention period rather than concentrated in a limited number of weeks, and usual-care participants with available primary outcome data and no major protocol deviations.

Unstandardized coefficients with 95% confidence intervals (CIs) will be used to estimate marginal means and group contrasts over time, which will be reported as mean differences (MDs). Standardized effect sizes, including Cohen’s d when appropriate, will be reported as complementary measures (69). For categorical outcomes, between-group effects will be estimated using risk differences (RDs) and relative risks (RRs), with 95% confidence intervals. When clinically interpretable, the number needed to treat, or harm, will be calculated from absolute risk differences. Effect sizes for differences in proportions will be estimated using Cohen’s *h*.

Model diagnostics will be examined to assess distributional assumptions, heteroscedasticity, and influential observations. If substantial departures are detected, robust linear mixed-effects models preserving the same fixed- and random-effects structure as the primary models will be considered. Sensitivity analyses may include restricted longitudinal data analysis under a common-baseline assumption of baseline variables showing clinically relevant imbalance between groups (70). Effect modification by sex, pubertal status, insulin delivery modality, insulin type, and CGM system will be explored by adding interaction terms to the primary model. These analyses will be considered exploratory.

Other exploratory analyses: Exploratory analyses will examine whether the magnitude of the intervention effect varies according to intervention exposure, adherence, and training-related characteristics. Dose–response analyses will use app-derived indicators of intervention exposure, including training frequency, completion of prescribed sessions, accumulated training volume, session duration, active and recovery times, training time, training type, and, when available, heart-rate metrics or perceived exertion. We will also explore whether within-participant changes in the primary and secondary outcomes are associated with changes in other clinical, behavioral, and physical-fitness variables. These change–change associations will be examined using regression models adjusted for treatment group, baseline values, hospital, and other relevant prespecified covariates. All such analyses will be considered exploratory and will not be interpreted as providing evidence of causal relationships.

Moreover, participants will be classified as responders or non-responders according to prespecified thresholds based on clinical guidelines (38), or minimally relevant standardized effect sizes (71), with the direction of beneficial response defined separately for each outcome. Response prevalence will be calculated by treatment group and compared using the χ² test. Additional longitudinal models may include responder status interaction to explore whether intervention effects differ between responder categories. To identify baseline profiles associated with response, exploratory regression models will be fitted. These analyses will be considered hypothesis-generating.

### 2.16 Frequency and plans for auditing trial conduct

Trial conduct will be reviewed weekly by the coordinating team using the Diactive-1 web-based platform to ensure protocol fidelity, data quality, participant safety, and compliance with ethical and regulatory requirements. Targeted virtual or on-site monitoring visits may be conducted if relevant protocol deviations, safety concerns, or site-specific performance issues are identified. Routine external audits are not planned given the low-risk nature of the intervention, although the sponsor, ethics committee, or relevant institutional audit unit may conduct independent audits in accordance with applicable regulations and institutional policies.

## 3 Discussion

This protocol addresses a practical gap in pediatric type 1 diabetes care: how to operationalize guideline-consistent resistance exercise and exercise-related diabetes education through a scalable digital intervention embedded in routine clinical pathways (20, 72). By testing Diactive-1 across 11 pediatric endocrinology units over 12 weeks, the trial is designed to move beyond single-center efficacy signals and estimate whether a tailored mHealth-supported resistance-training model can improve muscular strength, while also characterizing diabetes-related, physical, mental, and social health outcomes. Selecting handgrip strength as the primary outcome is methodologically coherent with the intervention target and clinically relevant because youth fitness assessment has standardized European reference values and muscular fitness in youth with type 1 diabetes has been associated with cardiometabolic risk (73–75). Nevertheless, the trial should be interpreted as testing a structured self-management and resistance-training strategy rather than an isolated exercise-dose effect, because the app combines training prescription, glucose-related educational prompts, tutorial support, and gamification (59).

Current recommendations support regular muscle-strengthening activities for children and adolescents with type 1 diabetes (20), whereas consensus statements emphasize that exercise benefits require individualized planning because glycemic responses vary by insulin availability, carbohydrate intake, technology use, and exercise characteristics (11, 29). This rationale is biologically plausible because skeletal muscle is a major metabolically active and endocrine tissue that secretes myokines involved in substrate oxidation, inflammation, angiogenesis, extracellular-matrix remodeling, and inter-organ crosstalk, while muscle contraction activates insulin-independent glucose uptake through GLUT-4 translocation and AMP-activated protein kinase pathways that may persist for several hours after exercise (76, 77). Prior randomized evidence suggests that exercise training can improve glycemic stability in youth with type 1 diabetes, but the literature remains heterogeneous regarding exercise type, duration, intensity, supervision, and implementation context, particularly for resistance-focused interventions (22). Diactive-1 has previously shown high usability and favorable single-center effects on insulin requirements, glycemic stability, muscular strength, lean mass, and bone-related outcomes, but those findings do not establish transportability across heterogeneous clinical settings, technologies, and participant profiles (10, 24). The present scale-up trial therefore has value primarily as an external-validity and implementation test of a refined intervention model rather than as a mere replication of the original RCT.

Major methodological strengths include the multicenter design, centralized computerized randomization with allocation concealment after baseline data entry, a comparator aligned with usual clinical care, and blinded outcome processing by the coordinating and statistical team. By bringing handgrip strength assessment into routine outpatient visits, the trial also explores the clinical feasibility of incorporating a functional marker of youth fitness into pediatric type 1 diabetes care, beyond traditional glycemic and cardiometabolic monitoring. The intervention is well specified through algorithmic progression based on handgrip-strength percentiles, prespecified training modalities, app-recorded exposure variables, glucose thresholds for session eligibility, and CGM- or AID-specific educational messages, which strengthens reproducibility and later interpretation of fidelity, adherence, and dose-response findings. The combination of clinical outcomes, glycemic technology, subjective barriers, physical literacy, quality of life, diet, and psychological outcomes broadens interpretability by linking effectiveness with mechanisms, user experience, and behavioral implementation. Embedding the study in routine pediatric endocrinology outpatient care and real-world daily environments should improve ecological validity, although this pragmatism also increases exposure to site-level heterogeneity and co-intervention variability. Importantly, the multicenter design will also provide information on the feasibility of implementing Diactive-1 across hospitals with different clinical workflows, which is essential for future scalability within public healthcare settings.

The main anticipated limitations are the impossibility of participant and clinician blinding, the use of self-reported instruments for several outcomes, heterogeneity in CGM and insulin-delivery systems, and incomplete objective heart-rate monitoring because this measure is planned only in a randomly selected hospital subsample. In participants without heart-rate monitoring, app-recorded session completion may overestimate actual intervention exposure, as the app can confirm that a session was started and completed but cannot fully verify whether all prescribed exercises were performed as intended. In addition, the app-based and home-based nature of the intervention may introduce variability in exercise execution, engagement, and access related to device availability, connectivity, or family support (78). Although our previous single-center RCT showed improvements within 12 weeks and sustained benefits for some outcomes up to 24 weeks (10, 24), the present 12-week multicenter trial will primarily assess short-term effectiveness and implementation across diverse clinical settings. It will not determine longer-term durability, post-trial adoption, or whether early changes translate into sustained cardiometabolic or psychosocial benefits. Because usual care permits routine treatment changes and habitual physical activity, between-group contrasts will estimate the added value of Diactive-1 over pragmatic clinical care rather than the biological effect of resistance training under controlled laboratory conditions. Exploratory subgroup, responder, and dose-response analyses may help explain heterogeneity but should be interpreted as hypothesis-generating because the protocol is powered for the primary outcome rather than for interaction effects or multiple secondary endpoints. In the Spanish context, the influence of the technological eligibility criteria on representativeness is expected to be limited because sensor-based glucose monitoring is publicly funded for eligible pediatric patients with type 1 diabetes, compatible devices may be personal or loaned, and Internet availability is widespread. Nevertheless, transferability to settings with more limited access to glucose-monitoring technology or reliable Internet connectivity may be lower.

If the intervention proves effective and acceptable, the findings could support integration of digitally assisted resistance-training education into pediatric type 1 diabetes care pathways, particularly where exercise counseling is constrained by time, access to specialists, or uncertainty around glycemic management. By incorporating standardized handgrip strength assessment into routine outpatient visits, the trial may also support the use of simple functional measures alongside conventional glycemic and cardiometabolic monitoring. Equally, null or mixed findings would be informative because app-derived data could help determine whether limited effects reflect insufficient exposure, weak implementation, outcome insensitivity, or a true lack of incremental benefit over usual care.

## Data Availability

No datasets were generated or analyzed for this study protocol. Data generated during the trial will be made available in accordance with applicable ethical, legal, and data-protection requirements. Further inquiries may be directed to the corresponding author.
